# Synthesis and *in Vitro* Antimicrobial Activity of Some Pyrazolyl-1-carboxamide Derivatives

**DOI:** 10.3390/molecules16097736

**Published:** 2011-09-09

**Authors:** Essam Mohamed Sharshira, Nagwa Mohamed Mahrous Hamada

**Affiliations:** 1 Department of Chemistry, Faculty of Science, Alexandria University, Alexandria 426, Egypt; 2 Department of Chemistry, Faculty of Education, Alexandria University, Alexandria 21526, Egypt; Email: nagwahamada2002@yahoo.com

**Keywords:** chalcones, hydrazones, pyrazoles, pyrazolines

## Abstract

A series of 3,5-disubstituted pyrazole-1-carboxamides were obtained by treatment of chalcones with semicarbazide hydrochloride in dioxane containing sodium acetate/acetic acid as a buffer solution. N-acetyl derivatives of pyrazole-1-carboxamides were isolated in good yields either by treatment of the carboxamide derivatives with acetic anhydride or refluxing chalcones with semicarbazide in ethanol containing few drops of acetic acid to give the corresponding hydrazones. Subsequent treatment of hydrazones with acetic anhydride gave the desired N-acetyl pyrazole-1-carboxamides derivatives. When chalcones were refluxed with dioxane containing few drops of acetic acid, 4,5-dihydropyrazole-1-carboxamides were isolated, which were subsequently oxidized using 5% sodium hypochlorite in dioxane to afford pyrazole-1-carboxamides. The structures of isolated compounds were confirmed by elemental analyses and spectral methods. The isolated compounds were tested for their antimicrobial activities.

## 1. Introduction

Pyrazoles are an important class of five-membered heterocyclic compounds and were found to have potential antimicrobial [1,2,3], anti-inflammatory [4], antipyretic [5], antidepressant [6,7], tranquillizing [8], anticancer [9,10], antiviral [11], antihypertensive [12], antiarrhythmic [13], antitubercular [14], psychoanaleptic [15], anticonvulsant [16] and antidiabetic [17] activities. In view of this and our continued interest in the synthesis of pyrazoles [1,2,18,19], it was thought of interest to synthesize some new pyrazole derivatives starting from chalcone and semicarbazide [16,20].

## 2. Results and Discussion

**Scheme 1:**
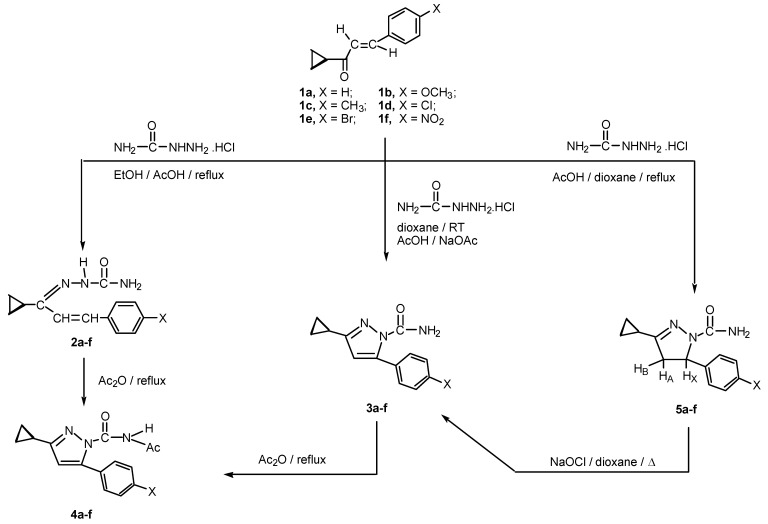
Synthesis of **2a-f**, **3a-f**, **4a-f** and **5a-f**.

The synthetic routes to our prepared compounds are shown in Scheme 1. The starting chalcones **1a-f** were prepared in good yields by conventional Claisen-Schmidt condensation by reacting appropriately substituted benzaldehydes and cyclopropylmethyl ketone in the presence of a base [1,21]. 

The method is attractive since it specifically generates the (*E*)-isomers of the products [22]. In this paper we show that reaction of chalcones **1a-f** with semicarbazide under different reaction conditions can affect the type of the product obtained and reaction pathways. For example, refluxing of chalcones **1a-f** with semicarbazide hydrochloride in ethanol containing acetic acid gave the corresponding semicarbazones **2a-f**. The structures of the isolated compounds were determined by IR and ^1^H-NMR spectra. The IR of the new semicarbazones revealed characteristic bands for vinyl CH=CH at 1,597–1,608, C=N at 1,627–1,663, C=O at 1,660–1,671, primary and secondary amines at 3,390–3,411 and 3,222–3,240 cm^−1^. The ^1^H-NMR spectra showed the presence of two broad exchangeable singlets at δ = 9.37–10.31 ppm, δ = 10.42–10.82 ppm characteristic for the NH_2_ and NH protons, respectively. A multiplet at δ = 7.12–7.89 ppm characteristic for the aromatic protons and the olefinic =C–*CH*=CH, a doublet at δ = 6.77–6.93 ppm for the olefinic =C–CH=*CH* proton. The cyclopropyl ring protons appeared as two multiplets in the range δ = 1.63–2.67 ppm (CH) and δ = 0.69–1.41 ppm (2 CH_2_), respectively. When chalcones **1a-f** were stirred at room temperature with semicarbazide hydrochloride in dioxane containing acetic acid/sodium acetate buffer solution, pyrazole-1-carboxamides **3a-f** were obtained in good yields. The IR of **3a-f** revealed the characteristic bands for Ar–C=C at 1,587–1,617, C=N at 1,629–1,657 and amide carbonyl bands at 1,652–1,670 cm^−1^, while the ^1^H-NMR spectra showed a singlet at δ = 6.73–6.83 for the pyrazole-C_4_-H. The N-acetyl derivatives **4a-f** were obtained by two different methods. In the first method, pyrazoles **3a-f** were heated under reflux with acetic anhydride, while in the second one, semicarbazones **2a-f** were cyclized to N-acetylpyrazoles **4a-f** using acetic anhydride. The ^1^H-NMR of **4a-f** exhibited a singlet of one proton intensity at δ = 6.75–6.87 ppm and another singlet of 3 protons intensity at δ = 2.11–2.18 ppm characteristic for pyrazole-C_4_–H and N–acetyl protons, respectively.

These results and the previous data reported in hydrazones derived from chalcones [1,2,23,24,25,26,27] showed that the substituent (G) attached to the hydrazono NH function (C=N–NH–G) plays a crucial role in changing reaction pathways and reaction products. For example, when G = aroyl group, cyclization occurs in the presence of acetic anhydride to give 1,3,4-oxadiazoles [1,23,24,25,26]. On the other hand, when G = carboxamide or aryl group, cyclization with acetic anhydride gave exclusive formation of pyrazole derivatives [2,27,28]. Finally, treatment of chalcones **1a-f** with semicarbazide hydrochloride in dioxane containing few drops of acetic acid gave pyrazolines **5a-f** in good yields.

The IR of **5** showed the presence of bands characteristic for an amide function at 1,657–1,679 (C=O) and 3,387–3,403 cm^−1^ (NH_2_). The pyrazoline ring CH_2_ protons resonated as a pair of doublets of doublets at δ = 3.07–3.17 ppm and δ = 3.71–3.86 ppm. The CH protons (H_X_) appeared as s doublet of doublets at δ = 5.37–5.45 ppm due to vicinal coupling with the two magnetically non-equivalent protons of the methylene group at position 4 of the pyrazoline ring (*J*_AB_ = 16 Hz, *J*_AX_ = 3.6 Hz, *J*_BX_ = 12 Hz). Finally, the structure of pyrazoles **3a-f** were confirmed by preparation through an alternative route via oxidation of pyrazoline **5a-f** using NaOCl/dioxane. The structures were verified by m.p. and mixed melting point experiments. The structures of all isolated compounds were confirmed by spectral and elemental analyses methods (Table 1 and Table 2).

### 2.1. Antimicrobial Activity

The *in vitro* antimicrobial activities of the newly synthesized compounds **3-5** were assayed against four test organisms (*Staphylococcus aureus* ATCC6538P, *Escherichia coli* ATCC8739, *Pseudomonas aeruginosa* ATCC9027 and *Candida albicans* ATCC2091) following the agar well-diffusion method [29] and using rifampicin (5 μg/disc) and ampicillin (10 μg/disc) as standard drugs. The tested compounds showed no significant effect against *Pseudomonas aeruginosa, *whereas they showed potent activity against *Staphylococcus aureus*, *Escherichia coli* and *Candida albicans.*

**Table 1:** Physical and Analytical Data of Compounds **2a-f, 3a-f, 4a-f,** and **5a-f**.

Compound	X	Yield (%)	M.P.°C	Molecular Formula	Calculated %			Found %		
					C	H	N	C	H	N
**2a**	H	76	161	C_13_H_15_N_3_O	68.12	6.55	18.34	68.06	6.49	18.31
**2b**	OCH_3_	82	171	C_14_H_17_N_3_O_2_	64.86	6.56	16.22	64.85	6.48	16.19
**2c**	CH_3_	80	174	C_14_H_17_N_3_O	69.14	7.00	17.28	69.09	6,97	17.31
**2d**	Cl	71	163	C_13_H_14_N_3_ClO	59.09	5.30	15.91	59.11	5.31	15.88
**2e**	Br	89	179	C_13_H_14_N_3_BrO	50.65	4.55	13.64	50.69	4.52	13.51
**2f**	NO_2_	93	182	C_13_H_14_N_4_O_3_	56.93	5.11	20.44	56.95	5.13	20.47
**3a**	H	77	193	C_13_H_13_N_3_O	68.72	5.73	18.50	68.77	5.69	18.49
**3b**	OCH_3_	66	183	C_14_H_15_N_3_O_2_	65.37	8.84	16.34	65.39	5.76	16.38
**3c**	CH_3_	67	176	C_14_H_15_N_3_O	69.71	6.22	17.43	69.75	6.19	17.44
**3d**	Cl	81	169	C_13_H_12_N_3_ClO	59.54	4.58	16.03	59.60	4.58	16.04
**3e**	Br	88	170	C_13_H_12_N_3_BrO	50.98	3.92	13.73	50.94	3.89	13.80
**3f**	NO_2_	91	199	C_13_H_12_N_4_O_3_	57.35	4.41	20.59	57.32	4.43	20.51
**4a**	H	69	188	C_15_H_15_N_3_O_2_	66.91	5.58	15.61	66.88	5.56	15.66
**4b**	OCH_3_	62	172	C_16_H_17_N_3_O_3_	64.21	5.69	14.05	64.19	5.66	14.08
**4c**	CH_3_	73	181	C_16_H_17_N_3_O_2_	67.84	6.01	14.84	67.91	6.03	14.89
**4d**	Cl	82	169	C_15_H_14_N_3_ClO_2_	59.21	4.61	13.82	59.17	4.62	13.78
**4e**	Br	71	176	C_15_H_14_N_3_BrO_2_	51.72	4.02	12.07	51.73	4.07	12.03
**4f**	NO_2_	79	197	C_15_H_14_N_4_O_4_	57.32	4.46	17.83	57.32	4.46	17.80
**5a**	H	71	152	C_13_H_15_N_3_O	68.12	6.55	18.34	68.16	6.49	18.36
**5b**	OCH_3_	62	149	C_14_H_17_N_3_O_2_	64.86	6.56	16.22	64.89	6.51	16.21
**2c**	CH_3_	69	143	C_14_H_17_N_3_O	69.14	7.00	17.28	69.17	6.97	17.29
**5d**	Cl	77	161	C_13_H_14_N_3_ClO	59.09	5.30	15.91	59.11	5.27	15.88
**5e**	Br	60	158	C_13_H_14_N_3_BrO	50.65	4.55	13.64	50.59	4.57	13.67
**5f**	NO_2_	77	169	C_13_H_14_N_4_O_3_	56.93	5.11	20.44	56.97	5.17	20.39

**Table 2:** IR and ^1^H-NMR Spectral Data of Compounds **2a-f, 3a-f, 4a-f, **and **5a-f**.

Comp.	IR cm^−^^1^ (KBr)				^1^H-NMR (δ / ppm) ^a^									
	VinylHC =CH orAr– C=C	C=N	C=O	NHand/orNH_2_	Ar-H’S and =C- *CH* =CH (m)	=C-CH=*CH *(d), J=12 Hz	Pyrazole C_4_–H (s)	Pyrazoline–H_A_dd,J_AX_ = 3.6Hz, dd, J_AB_ = 16Hz	Pyrazoline–H_B_ dd,J_AB_ = 16Hz, dd,J_BX_ = 12Hz	Pyrazoline–H_X_ dd,J_AX_ = 3.6Hz, dd,J_BX_=12Hz	NH and/or NH_2_ (s), D_2_O exchangeable	Cyclopropyl ring H’S		Ar–CH_3_, Ar–OCH_3_, CH_3_CO–(S)
												CH(m)	2(CH_2_) (m)	
**2a**	1603	1631	1664	3234 and 3402	7.31–7.76	6.77	–	–	–	–	10.11, 10.63	1.89–2.54	0.73–1.36	–
**2b**	1607	1633	1661	3235 and 3390	7.29–7.86	6.81	–	–	–	–	10.31, 10.57	1.83–2.36	0.72–1.38	3.66
**2c**	1597	1627	1669	3240 and 3401	7.26–7.74	6.79	–	–	–	–	9.37, 10.42	1.84–2.42	0.69–1.41	2.22
**2d**	1604	1645	1668	3227 and 3400	7.19–7.89	6.84	–	–	–	–	9.87, 10.73	1.79–2.41	0.75–1.36	–
**2e**	1608	1650	1660	3222 and 3409	7.17–7.77	6.87	–	–	–	–	9.91, 10.61	1.71–2.45	0.78–1.26	–
**2f**	1598	1663	1671	3228 and 3411	7.12–7.81	6.93	–	–	–	–	9.77, 10.82	1.63–2.67	0.77–1.31	–
**3a**	1597	1634	1652	3387	7.24–7.86 ^b^	–	6.83	–	–	–	10.54	1.76–2.53	0.74–1.34	–
**3b**	1593	1629	1660	3381	7.26–8.02 ^b^	–	6.74	–	–	–	10.61	1.81–2.33	0.71–1.36	3.71
**3c**	1587	1633	1665	3401	7.21–7.98 ^b^	–	6.82	–	–	–	10.33	1.87–2.39	0.67–1.39	2.29
**3d**	1591	1644	1659	3397	7.13–7.79 ^b^	–	6.81	–	–	–	10.39	1.66–2.43	0.69–1.32	–
**3e**	1617	1650	1655	3395	7.11–7.75 ^b^	–	6.73	–	–	–	10.31	1.72–2.49	0.66–1.33	–
**3f**	1614	1657	1670	3402	7.31–7.64 ^b^	–	6.79	–	–	–	10.70	1.70–2.62	0.71–1.29	–
**4a**	1607	1634	1659	3230	7.25–7.83 ^b^	–	6.84	–	–	–	9.39	1.77–2.54	0.72–1.31	2.11
**4b**	1602	1636	1660	3261	7.23–7.91 ^b^	–	6.75	–	–	–	9.29	1.79–2.55	0.73–1.37	2.13,3.69
**4c**	1601	1622	1663	3233	7.23–7.89 ^b^	–	6.81	–	–	–	9.30	1.82–2.41	0.70–1.36	2.13,2.21
**4d**	1598	1639	1662	3241	7.17–7.83 ^b^	–	6.86	–	–	–	9.27	1.69–2.43	0.72–1.32	2.14
**4e**	1603	1651	1663	3237	7.16–7.73 ^b^	–	6.77	–	–	–	9.23	1.71–2.53	0.69–2.39	2.17
**4f**	1611	1657	1676	3227	7.33–7.60 ^b^	–	6.87	–	–	–	9.37	1.73–2.59	0.73–2.58	2.18
**5a**	–	1635	1657	3398	7.21–7.79 ^b^	–	–	3.09	3.79	5.42	10.63	1.74–2.51	0.71–1.32	–
**5b**	–	1638	1659	3387	7.26–7.89 ^b^	–	–	3.11	3.71	5.45	10.65	1.76–2.53	0.76–1.36	3.67
**5c**	–	1627	1661	3403	7.22–7.87 ^b^	–	–	3.07	3.72	5.39	10.57	1.80–2.41	0.69–1.40	2.24
**5d**	–	1634	1667	3396	7.19–7.74 ^b^	–	–	3.13	3.81	5.44	10.49	1.72–2.47	0.76–1.45	–
**5e**	–	1660	1668	3391	7.15–7.77 ^b^	–	–	3.14	3.77	5.37	10.44	1.70–2.55	0.69–2.43	–
**5f**	–	1667	1679	3398	7.36–6.69	–	–	3.17	3.86	5.40	10.56	1.75–2.61	0.78–2.60	–

^a^ Solution in DMSO-d_6_; ^b^ The chemical shift only indicates Ar–H’s.

The maximum activity (+++; MIC = 25 μg/mL) was indicated for compounds **3d, 3e, 4f**, and **5f**. These results suggest that electron-withdrawing groups (X = Cl, Br and NO_2_) in the pyrazolyl compounds **3** play a crucial role in enhancing the activity. For *Staphylococcus aureus*, compounds **3a, 3b, 3c** and **3e** showed moderate activity (++; MIC = 50 μg /mL), while compounds **4a, 4b, 5a** and **5b** showed only slight activity (+; MIC = 75 μg/mL). Compounds **3b, 3c, 4d, 4e, 5d** and **5e** exhibited moderate activity against *Escherichia coli* whereas, compounds 3a, 3e and 4b showed a slight activity against this organism. Moreover, compounds **3b, 3c, 3e, 4a, 4d, 4e, 5b, 5d** and **5e** showed moderate activity against *Candida albicans*, whereas compounds **3a, 4b, 4c, 5a** and **5c** showed slight activity. In summary, all of the tested compounds showed antifungal activities, and compounds **3d, 3f, 4f** and **5f** were found to be the most active against all the tested microorganisms. The results are summarized in Table 3.

**Table 3:** Antimicrobial activities of newly synthesized compounds **3–5**.

Compound	X	*Sta staphylococcus*	*Escherichia coli*	*Candida albicans*
**3a**	H	++	+	+
**3b**	OCH_3_	++	++	++
**3c**	CH_3_	++	++	+ +
**3d**	Cl	+++	+++	+++
**3e**	Br	++	+	++
**3f**	NO_2_	+++	+++	+++
**4a**	H	+	−	++
**4b**	OCH_3_	+	+	+
**4c**	CH_3_	−	−	+
**4d**	Cl	++	++	++
**4e**	Br	++	++	++
**4f**	NO_2_	+++	+++	+++
**5a**	H	+	−	+
**5b**	OCH_3_	+	−	++
**5c**	CH_3_	+	−	+
**5d**	Cl	++	++	++
**5e**	Br	++	++	++
**5f**	NO_2_	+++	+++	+++

+++ for high activity (MIC = 25 μg/mL); ++ for moderate activity (MIC = 50 μg/mL); + for slight activity (MIC = 75 μg/mL) and − for inactive.

## 3. Experimental

### 3.1. General

Melting points were taken in open capillary tubes using an Electrothermal apparatus 9100 (UK) and are uncorrected. Microanalyses were performed at the Faculty of Science, Cairo University, Cairo, Egypt, using an Elementary Vario el III C, H, N, S Analyzer (Germany). IR spectra were recorded using potassium bromide disks on a Perkin-Elmer 1650 spectrophotometer (Faculty of Science, Alexandria University, Alexandria, Egypt). ^1^H-NMR spectra were determined on a Varian EM-390 MHz spectrophotometer, using TMS as internal standard. The biological activities were evaluated at the lab of microbiology, Faculty of pharmacy, Alexandria University, Alexandria, Egypt.

### 3.2. General Procedure for Preparation of E-1-Cyclopropyl-3-(p-substituted-phenyl)-2-propenones **1a-f**

To a cold solution of sodium hydroxide (3 g) in aqueous ethanol (50 mL, 60%), cyclopropylmethyl ketone (10 mmol) was added dropwise (30 min), while rapidly stirring, then the desired *p*-substituted benzaldehyde (10 mmol) was added dropwise (30 min). After five hours, the mixture was left overnight in refrigerator. The separated solid was filtered, washed with water and dried, then recrystallized from ethanol as colorless needles. The physical properties and all the spectral data were as reported in the literature [1,21].

### 3.3. General Procedure for Preparation of 1-Cyclopropyl-3-(p-substituted-phenyl)-2-propene-1-semicarbazones **2a-f**

A solution of chalcones **1a-f** (10 mmol) in ethanol (10 mL) was refluxed with the appropriate semicarbazide hydrochloride (10 mmol) in glacial acetic acid (2 mL) for about five hours, then the reaction mixture was poured onto crushed ice and was kept overnight at room temperature, the separated solid was filtered off, washed successively with water and dried, then recrystallized from methanol. Melting points, IR and NMR data: see Table 1 and Table 2.

### 3.4. General Procedure for Preparation of 3-Cyclopropyl-5-(p-substituted-phenyl)-pyrazole-1-carbox-amides **3a-f**

*Method A:* A solution of chalcones **1a-f** (10 mmol) in dioxane (10 mL) and semicarbazide (10 mmol) in sodium acetate/ acetic acid buffer solution [30]. The reaction mixture was stirred at room temperature for 24 hours. The separated solid was filtered off, washed with water, dried and recrystallized from methanol to give **3a-f**. Melting points, IR and NMR data: see Table 1 and Table 2.

*Method B:* A solution of the appropriate pyrazoline **5a-f** (10 mmol), dioxane (10 mL) and sodium hypochlorite (5 mL, 5%) was heated over a boiling water bath until effervescence occurs; heating was continued for a further 10 minutes. The reaction mixture was allowed to reach ambient temperature and the separated solid was filtered, washed with water, dried and recrystallized from methanol to give the corresponding pyrazoles **3a-f**. The physical properties and all the spectral data were identical with those prepared by method A.

### 3.5. General Procedure for Preparation of 3-Cyclopropyl-5-(p-substituted-phenyl)-pyrazole-1-(N-acetyl)-carboxamides **4a-f**

*Method A:* A mixture of the appropriate semicarbazone **2a-f** (10 mmol) and acetic anhydride (15 mL) was heated under reflux for three hours. After the reaction mixture attained room temperature, it was poured into crushed ice and the oily product deposited was decanted from water and extracted with ether. The ether layer was washed with sodium bicarbonate, followed by water, dried over anhydrous sodium sulphate and evaporated to give the corresponding pyrazoles **4a-f** as needles. Melting points, elemental analyses, IR and NMR data: see Table 1 and Table 2.

*Method B:* A mixture of pyrazoles **3a-f** (10 mmol) in acetic anhydride (5 mL) was heated under reflux for 30 minutes. The reaction mixture was treated as mentioned in method A to give the N–acetyl derivatives **4a-f**.

### 3.6. General Procedure for Preparation of 4,5-Dihydro-3-cyclopropyl-5-(p-substituted-phenyl)-pyrazole-1-carboxamides **5a-f**

A solution of chalcones **1a-f** (10 mmol) in dioxane (10 mL) was refluxed with the appropriate semicarbazide hydrochloride (10 mmol) in glacial acetic acid (1 mL) for 4 hours, then the reaction mixture was treated as mentioned for **2a-f**. Melting points and spectral data are listed in Table 1 and Table 2.

### 3.7. Determination of Antimicrobial Activity

All the synthesized heterocyclic compounds were tested against four different microorganisms: *Staphylococcus aureus*, *Escherichia coli*, *Pseudomonas aeruginosa* and *Candida albicans*. The agar well-diffusion method was applied for the determination of inhibition zones and minimum inhibitory concentrations (MICs). Briefly, broth culture (0.75 mL) containing *ca. *10^6^ colon-forming units (CFU) per mL of the test strain was added to nutrient agar medium (75 mL) at 45 °C, mixed well, and then poured into a 15 cm sterile metallic Petri plate. The medium was allowed to solidify, and 8 mm wells were dug with a sterile metallic borer. Then, a DMSO solution of the test sample (1 mL, 1 mg/mL) was added to the respective wells. DMSO served as negative control, and the standard antimicrobial drugs rifampicin (5 μg/disc) and ampicillin (10 μg/disc) were used as positive controls. Triplicate plates of each microorganism strain were prepared and were incubated aerobically at 37 °C for 24 h. The activity was determined by measuring the diameter of zone showing complete inhibition (mm), thereby, the zones were precisely measured with the aid of a Vernier Caliper (precision 0.1 mm). The growth inhibition was calculated with reference to the positive control.

## 4. Conclusions

This work describes different methods for the synthesis of new heterocyclic pyrazole derivatives. The antimicrobial activity of these compounds was evaluated against Gram-positive, Gram-negative bacteria and fungi. Most of the compounds showed moderate antimicrobial activity.
